# Supplementary material for: The Transcriptomic Landscape of Pediatric Astrocytoma
Source: Int J Mol Sci. 2022 Oct 21;23(20):12696. doi: 10.3390/ijms232012696 (PMC9604090; doi:10.3390/ijms232012696)
Supplement: Supplementary file 1 [file ijms-23-12696-s001.zip › Supp_Fig_legends.pdf]

The transcriptomic landscape of pediatric astrocytoma

Supplementary figure legends

Supplementary Figure S1. Quality control of the reads. A) total read counts throughout the 20 samples used in this work. B) Mean quality scores of the reads in all samples.

Supplementary Figure S2. Quality control of mapped reads and library size distribution. a) Percentages of mapped reads are displayed in blue for uniquely mapped reads, green for ribosomal RNA mapped reads and gray for non-chromosomal RNA mapped reads (e.g., mitochondrial, sequence contigs not yet mapped on chromosomes). b) Boxplot displaying the distribution of library size before (left) and after (right) filtering and normalization.

Supplementary Figure S3. Schematic representation of the comparisons between each grade of pediatric astrocytoma and matching healthy tissue.

Supplementary Figure S4. Comparison of DE genes in this and another study. a) Venn diagram showing overlap in the number of genes upregulated (left) and downregulated (right) for pilocytic astrocytoma in this study, Rorive et al. [16] and Seifert et al. [17].

Supplementary Figure S5. **ssGSEA scores.** a) Heatmap with hierarchical clustering of the ssGSEA enrichment scores for the genes in the transcriptional signature projected in the RNAseq data of pediatric astrocytomas. The transcriptional signatures for cerebellar PA (14 genes), cerebellar anaplastic astrocytoma (17 genes), glioblastoma (20 genes) and healthy cerebrum and cerebellum (17 genes) are color-coded and depicted in the right side of the heatmap, whereas their grade is depicted on the top of the heatmap. b) Heatmap with hierarchical clustering of the ssGSEA enrichment scores for the genes in the transcriptional signature projected in the publicly available GEO dataset GSE5675 with the transcriptional profile of 41 pediatric PAs in different locations of the brain.
