# Supplementary material for: The Transcriptomic Landscape of Pediatric Astrocytoma
Source: Int J Mol Sci. 2022 Oct 21;23(20):12696. doi: 10.3390/ijms232012696 (PMC9604090; doi:10.3390/ijms232012696)
Supplement: Supplementary file 1 [file ijms-23-12696-s001.zip › Supplementary_Tables_S2-S4.pdf]

Supplementary Table S2. The top 10 and other representative annotation clusters for the up- and down regulated genes in the different degrees of pediatric astrocytoma

Annotation cluster	Representative annotation terms	Enrichment score
	**Up regulated biological functions in PA from Cerebellum**	
**1***	Immunity and Innate immune response*	24.73
**2**	Glycoprotein and signal peptide	23.12
**3**	Membrane, transmembrane helix and integral component of membrane	14.8
**4**	Lysosome and Endosome	9.36
**5**	Staphylococcus aureus infection, Phagosome, interferon-gamma-mediated signaling pathway	7.84
**6**	B cell receptor signaling pathway	4.94
**7**	Pleckstrin homology-like domain	4.37
**8**	Immunoglobulin domain	3.97
**9**	Regulation of small GTPase mediated signal transduction	3.56
**10**	Natural killer cell mediated cytotoxicity	3.45
**68* (Table 2)**	Positive regulation of MAP kinase activity* (Described in table 2)	0.76
	**Down regulated biological functions in PA from Cerebellum**	
**1***	Synapse and cell junction*	16.65
**2**	Nucleosome core and histone-fold	3.96
**3**	transmembrane helix and integral component of membrane	2.96
**4***	Neurotransmitter-gated ion-channel, conserved site*	2.73
**5**	Sterile alpha motif domain	2.54
**6***	Neurogenic differentiation factor and Transcription factor, basic helix-loop-helix*	2.35
**7**	Histone H4, DNA replication-dependent nucleosome assembly, telomere organization, negative regulation of gene expression, epigenetic, gene silencing by RNA	2.25
**8**	Histone H2B	2.24
**9**	Ankyrin repeat-containing domain	2.04
**10**	Gamma-aminobutyric acid signaling pathway	1.95

	**Up regulated biological functions in grade II astrocytoma**	
**1**	Membrane, transmembrane helix and integral component of membrane	2.4
**2**	Immunity and Innate immune response	2.18
**3**	Immunoglobulin/major histocompatibility complex, conserved site	2.15
**4**	Pathways in cancer, Glioma, MicroRNAs in cancer	1.66
**5**	Cytokine activity	1.55
**6**	Immunoglobulin domain	1.51

**7**	Extracellular region and secreted proteins	1.43
**8**	Positive regulation of transcription from RNA polymerase II promoter and negative regulation of cell proliferation	1.33
**9**	Cyclins and Cell Cycle Regulation	1.23
**10**	Pleckstrin homology-like domain	1.1

	**Up regulated biological functions in Glioblastoma**	
**1****	Sister chromatid cohesion, Centromere and Kinetochore**	16.2
**2**	ATP-binding and nucleotide phosphate-binding region:ATP	7.38
**3**	Collagen and protein digestion and absorption	5.53
**4**	kinesin complex and microtubule motor activity	5.36
**5**	EGF-like calcium-binding, Epidermal growth factor-like domain	4.09
**6**	IQ motif, EF-hand binding site	3.84
**7**	Nucleosome core and Histone-fold	3.79
**8**	Laminin G domain	3.6
**9**	DNA helicase activity	3.07
**10**	Histone H2A and chromatin silencing	2.88
	****Sub clustering of annotation cluster 1**	
**1**	Centromere and Kinetocore	45.05
**2**	Mitotic nuclear division	29.02
**3**	Spindle microtubule and protein phosphorylation	4.32
**4**	Spindle pole and Oocyte meiosis	4.04
**5**	Chromosome passenger complex and protein sumoylation	4.01
**6**	Microtubule and kinesin complex	3.09
**7**	mitotic spindle assembly checkpoint, regulation of chromosome segregation and cell cycle	2.25
**8**	Apoptosis and protein phosphorylation	1.19
	**Down regulated biological functions in Glioblastoma**	
**1**	Synapse, cell junction and postsynaptic cell membrane	27.26
**2**	Ion transport and Voltage-gated channel	10.84
**3**	Calmodulin-binding	8.42
**4**	Morphine addiction and GABAergic synapse	7.78
**5**	Transmembrane helix and integral component of membrane	7.77
**6**	SH3 domain and Src homology-3 domain	6.36
**7**	cGMP-PKG signaling pathway and renin secretion	5.15
**8**	Regulation of exocytosis and SNARE binding	4.05
**9**	PDZ domain	3.48

**10**	C2 calcium-dependent membrane targeting, calcium ion-regulated exocytosis of neurotransmitter and positive regulation of dendrite extension	3.47

**Down regulated biological functions shared between anaplastic astrocytoma and Glioblastoma**		
**1**	Pleckstrin homology-like domain	1.33
**2**	Synapse and cell junction	1.16
**3**	Cytoplasmic vesicle	1
**4**	Integral component of membrane	0.08

The clusters are ordered by group enrichment score. The representative biology terms associated with the annotation clusters were manually selected. The clusters are ordered by group enrichment score and thus by their biological significance. Single Asterisk indicate biological pathways previously reported to be altered in this subtype of astrocytoma. Double asterisks represent cluster than have been sub cauterized.

Supplementary Table S3: Six genes from the cluster 68 of the up regulated biological functions in PA from Cerebellum, involved in the positive regulation of the MAP kinase activity

Gene name (Symbol)	LogFC	P-value
Phosphoinositide-3-kinase regulatory subunit 5 (PIK3R5)	4.3	1.81E-05
Phosphatidylinositol-4,5-bisphosphate 3-kinase catalytic subunit gamma (PIK3CG)	3.7	0.00051182
Leucine rich repeat kinase 2 (LRRK2)	3.3	0.00028082
Transforming growth factor beta 1 (TGFB1)	2.7	0.00108505
Platelet derived growth factor subunit B (PDGFB)	2.5	0.002051454
IQ motif containing GTPase activating protein 1 (IQGAP1)	2	0.001865071

Supplementary Table S4. Annotations of mis regulated genes in the different degrees of pediatric astrocytoma

Gene	Representative annotation terms
**Down regulated genes in grade II astrocytoma**	
**PPFIA4**	Neurotransmitter secretion, glutamate secretion,
**SLF1**	DNA replication initiation, DNA repair, cellular response to DNA damage stimulus, mitotic G2 DNA damage checkpoint
**GFOD2**	Extracellular matrix organization, oxidation-reduction process,
**MPP3**	Signal transduction, GMP metabolic process, GDP metabolic process

**Up regulated genes in Anaplastic astrocytoma (Cerebellum)**	
**PFKFB2**	Fructose and mannose metabolism, AMPK signaling pathway, Thyroid hormone signaling pathway
**CACNA2D4**	MAPK signaling pathway
**TGFB2**	MAPK signaling pathway, Cytokine-cytokine receptor interaction, FoxO signaling pathway, Cell cycle, TGF-beta signaling pathway, Pathways in cancer, Proteoglycans in cancer, MicroRNAs in cancer, Colorectal cancer, Renal cell carcinoma, Pancreatic cancer, Chronic myeloid leukemia
**HLA-F**	Endocytosis, Phagosome, Cell adhesion molecules (CAMs), Antigen processing and presentation, HTLV-I infection, Herpes simplex infection, Epstein-Barr virus infection, Viral carcinogenesis, Autoimmune thyroid disease,
**Down regulated genes in Anaplastic astrocytoma (Cerebellum)**	
**MAX**	MAPK signaling pathway, Pathways in cancer, Transcriptional misregulation in cancer, Small cell lung cancer
**GLS2**	Arginine biosynthesis, Alanine, aspartate and glutamate metabolism, D-Glutamine and D-glutamate metabolism, Metabolic pathways, Glutamatergic synapse, GABAergic synapse, Proximal tubule bicarbonate reclamation, MicroRNAs in cancer, Central carbon metabolism in cancer
**MAML3**	Notch signaling pathway
**ALS2**	Amyotrophic lateral sclerosis (ALS)

**Up regulated genes shared between Anaplastic astrocytoma and Glioblastoma**	
**NCAPH**	Mitotic chromosome condensation, cell division,
**B3GNT9**	Protein glycosylation in Golgi membrane
**TMEM45A**	Integral component of membrane

**Up regulated genes unique for Anaplastic astrocytoma (vs. Glioblastoma)**	

**CACNA2D4**	MAPK signaling pathway, Regulation of ion transmembrane transport
**TGFB2**	MAPK signaling pathway, cell cycle, TGF-beta signaling pathway, pathways in cancer, proteoglycans in cancer, MicroRNAs in cancer
**EMILIN2**	Cell division and chromosome partitioning
**PFKFB2**	Fructose and mannose metabolism, AMPK signaling pathway, Thyroid hormone signaling pathway,
**UAP1L1**	Amino sugar and nucleotide sugar metabolism, Metabolic pathways, Biosynthesis of antibiotics
**F13A1**	Complement and coagulation cascades
**DPYD**	Pyrimidine metabolism, beta-Alanine metabolism, Metabolic pathways
**ERP27**	Protein folding, response to endoplasmic reticulum stress
**EPB41L2**	Cortical actin cytoskeleton organization, actomyosin structure organization
**HLA-F**	Endocytosis, Phagosome, Cell adhesion molecules (CAMs)
**MS4A4A/MS4A6A**	Integral components of membrane,

**Down regulated genes unique for Anaplastic astrocytoma (vs. Glioblastoma)**	
**ALS2**	Cell division and chromosome partitioning / Cytoskeleton
**GPR137C**	Integral component of membrane,
**MAX**	MAPK signaling pathway, Pathways in cancer, Transcriptional misregulation in cancer
**SLF1**	DNA replication initiation, DNA repair, cellular response to DNA damage stimulus
**MAML3**	Notch signaling pathway
