# Supplementary figures and images for: The Transcriptomic Landscape of Pediatric Astrocytoma
Source: Int J Mol Sci. 2022 Oct 21;23(20):12696. doi: 10.3390/ijms232012696 (PMC9604090; doi:10.3390/ijms232012696)

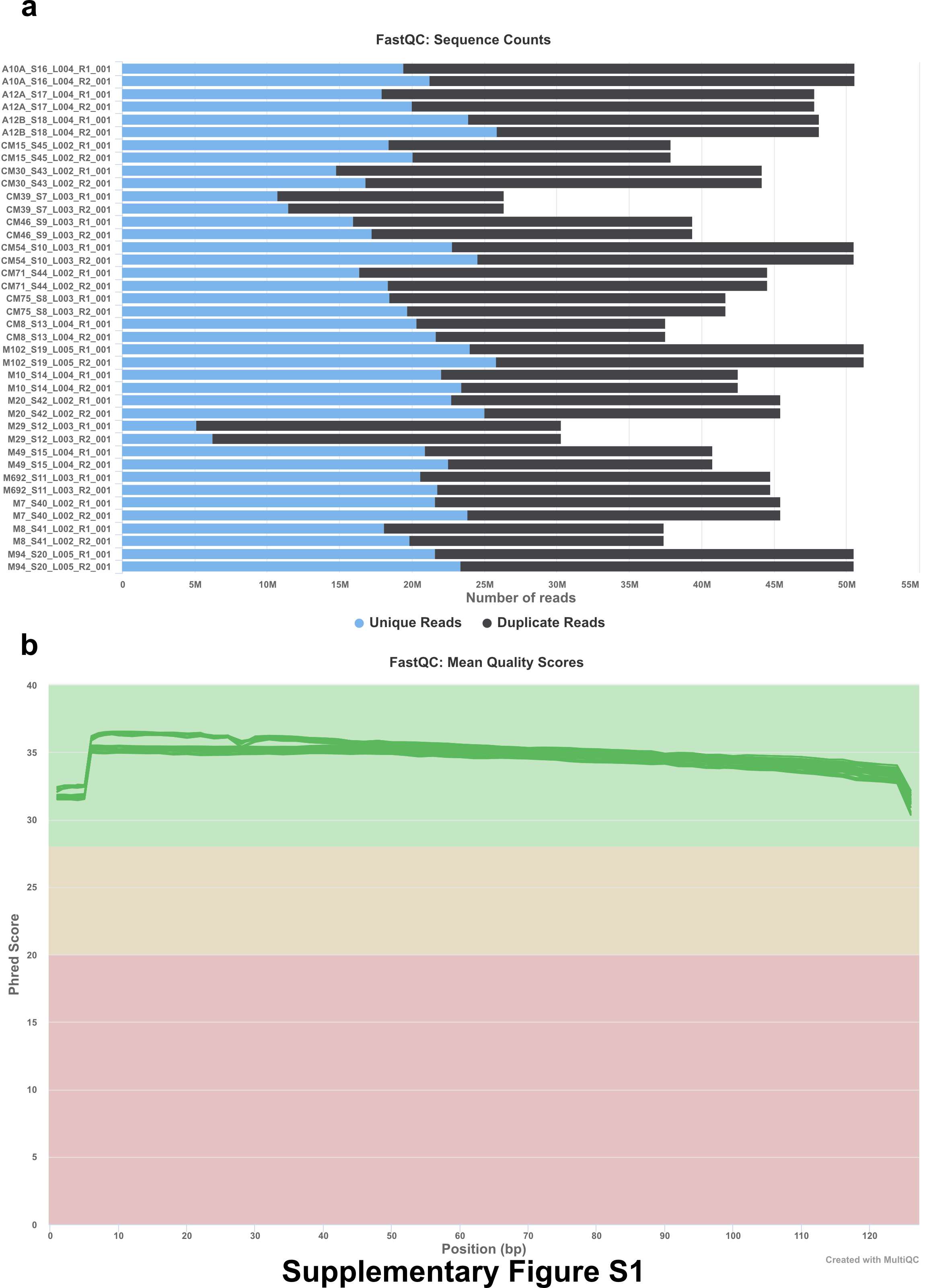

Supplement: Supplementary file 1 [file ijms-23-12696-s001.zip › FigS1.tiff]

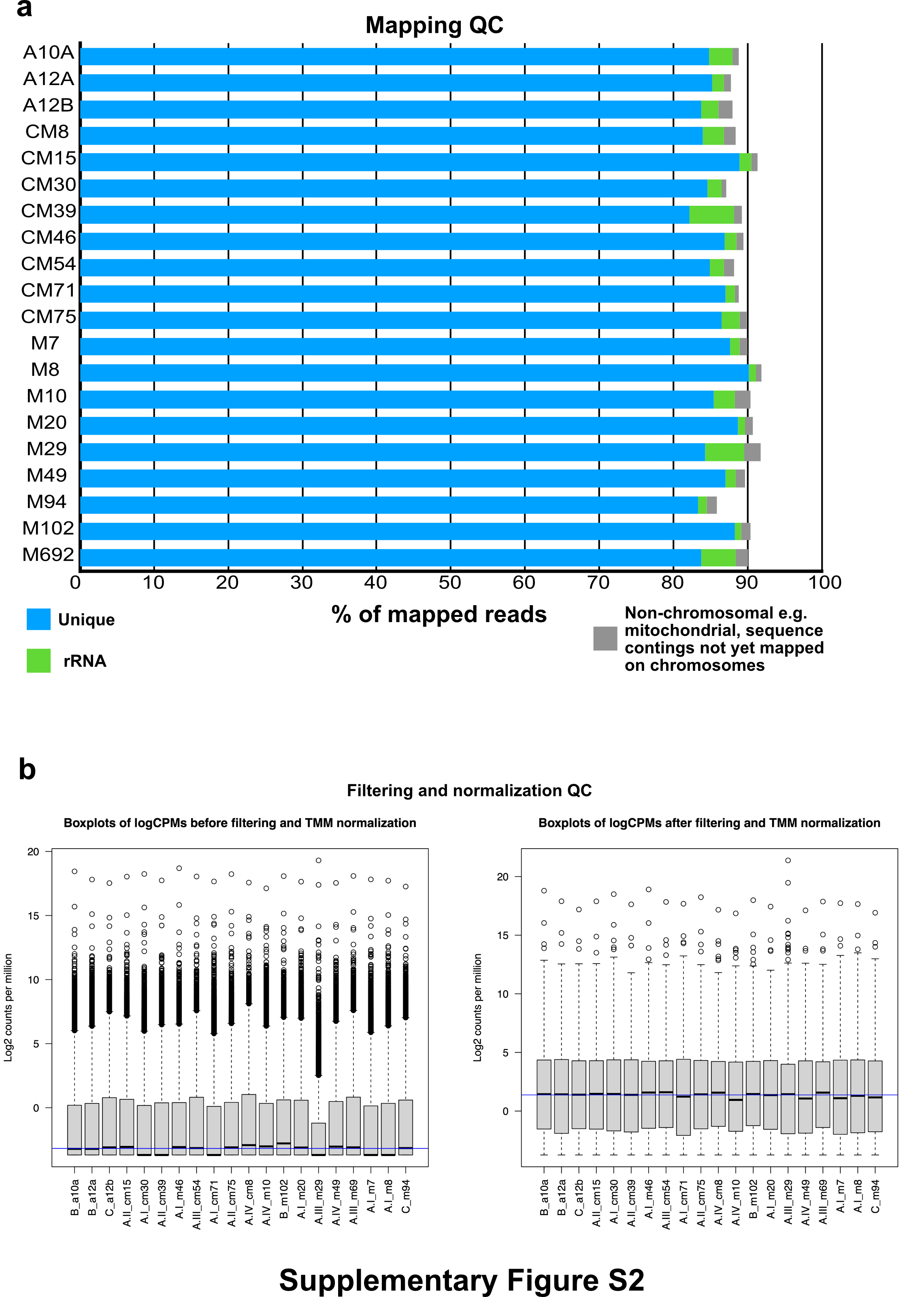

Supplement: Supplementary file 1 [file ijms-23-12696-s001.zip › FigS2.tiff]

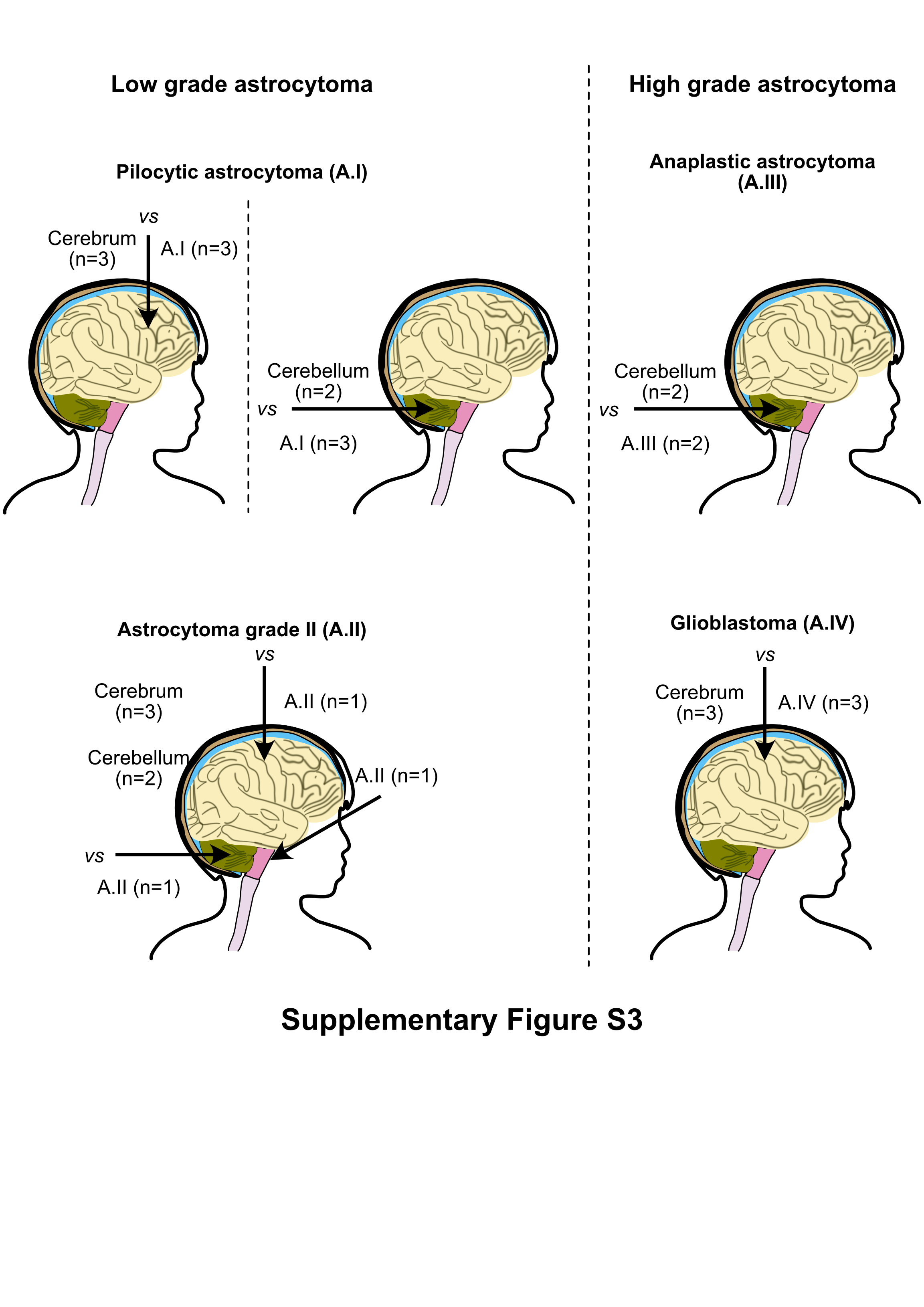

Supplement: Supplementary file 1 [file ijms-23-12696-s001.zip › FigS3.tiff]

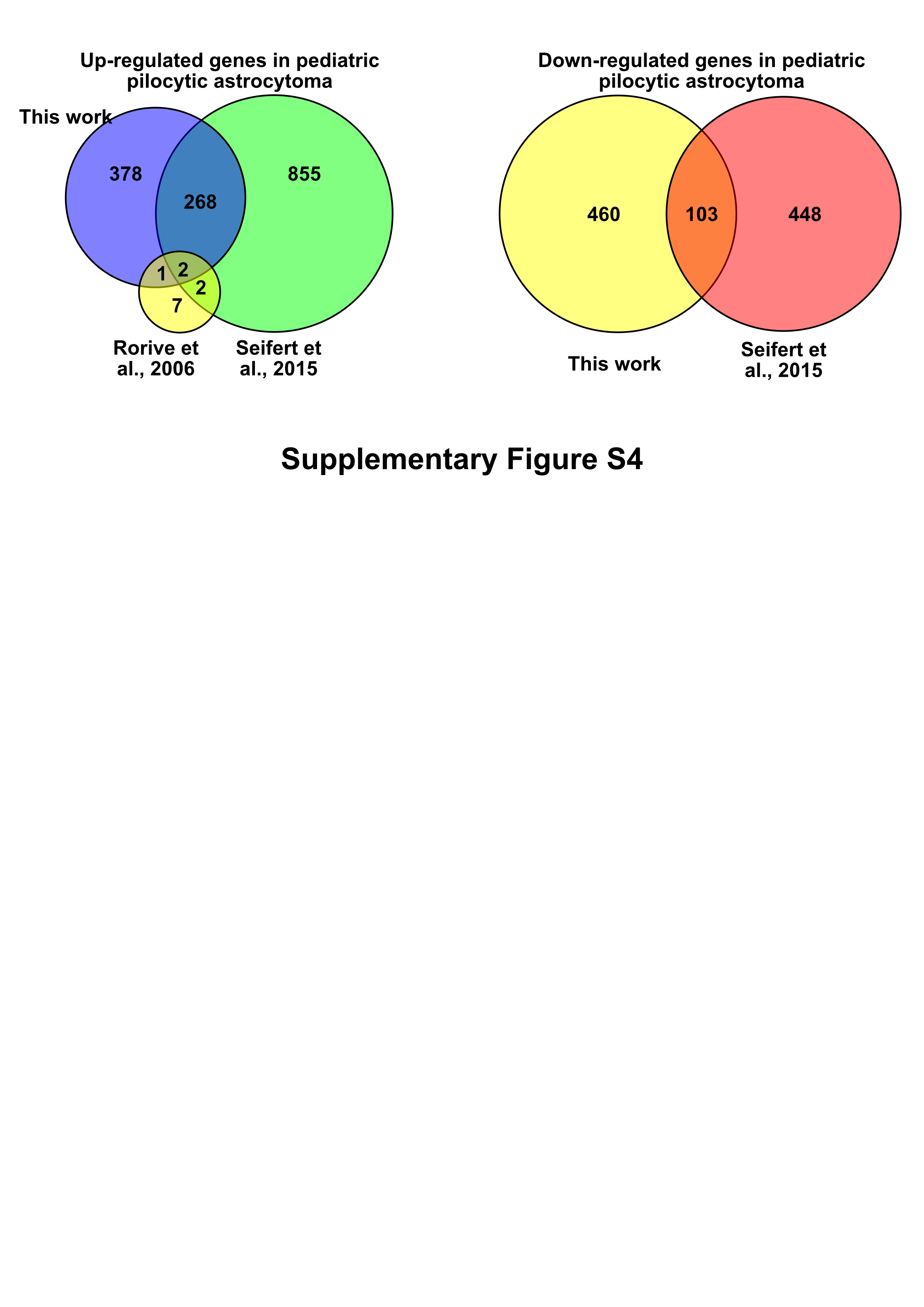

Supplement: Supplementary file 1 [file ijms-23-12696-s001.zip › FigS4.tiff]

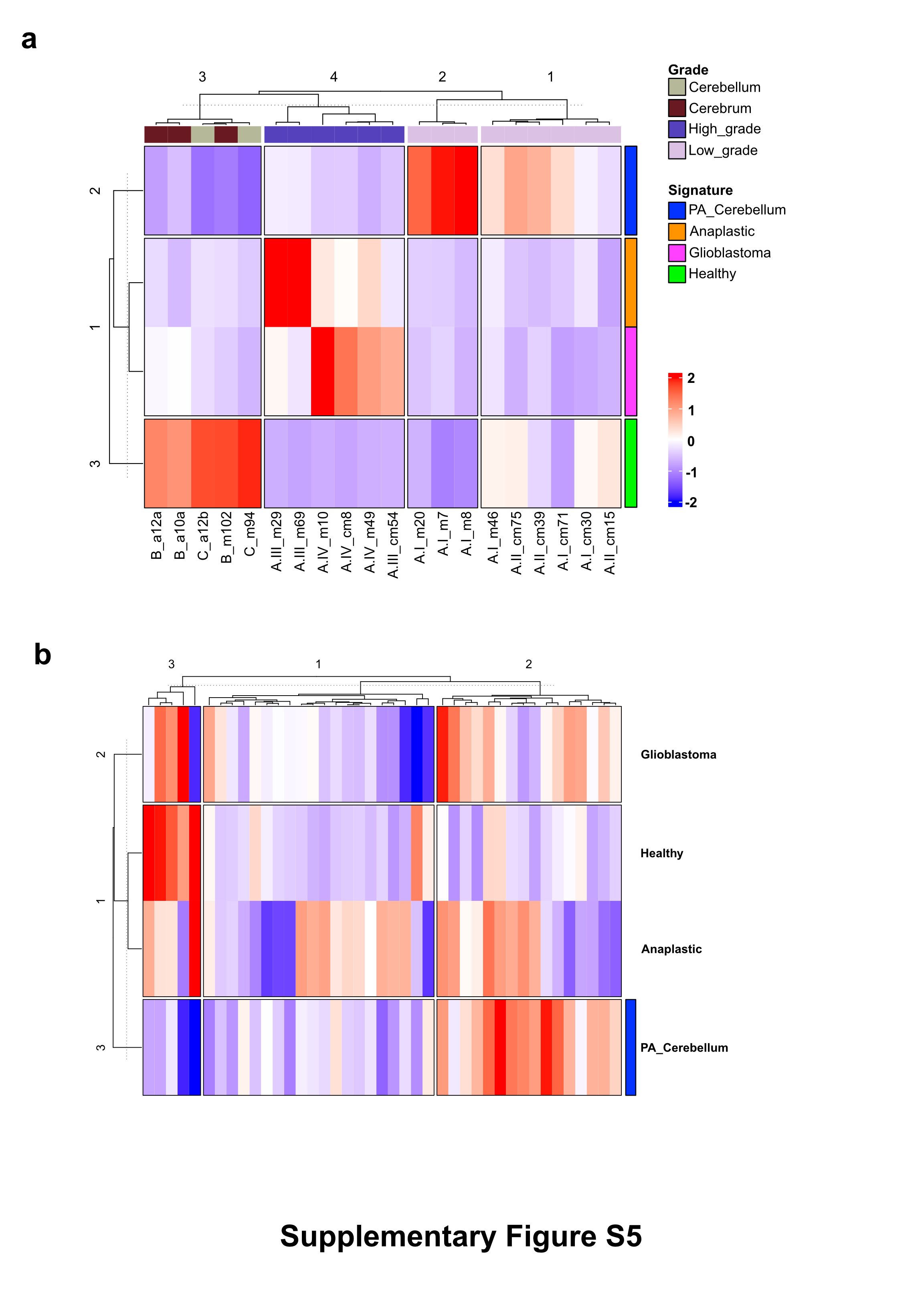

Supplement: Supplementary file 1 [file ijms-23-12696-s001.zip › FigS5.tiff]
